# The characterisation of *AOP2*: a gene associated with the biosynthesis of aliphatic alkenyl glucosinolates in *Arabidopsis thaliana*

**DOI:** 10.1186/1471-2229-10-170

**Published:** 2010-08-11

**Authors:** Calida S Neal, Dale P Fredericks, Cara A Griffiths, Alan D Neale

**Affiliations:** 1 School of Biological Sciences, Monash University, Melbourne, Victoria, 3800, Australia; 2 Dept of Gastroenterology/Renal Unit, School of Medicine, Flinders University, Adelaide, South Australia, 5042, Australia; 3 ARC Special Research Centre for Green Chemistry, Monash University, Melbourne, Victoria, 3800, Australia

## Abstract

**Background:**

Glucosinolates, a group of nitrogen and sulfur containing compounds associated with plant-insect interactions, are produced by a number of important *Brassicaceae *crop species. In *Arabidopsis *the *AOP2 *gene plays a role in the secondary modification of aliphatic (methionine-derived) glucosinolates, namely the conversion of methylsulfinylalkyl glucosinolates to form alkenyl glucosinolates, and also influences aliphatic glucosinolate accumulation.

**Results:**

This study characterises the primary structural variation in the coding sequences of the *AOP2 *gene and identifies three different *AOP2 *alleles based on polymorphisms in exon two. To help determine the regulatory mechanisms mediating *AOP2 *expression amongst accessions, *AOP2 *5' regulatory regions were also examined however no major differences were identified. Expression of the *AOP2 *gene was found to be most abundant in leaf and stem tissue and was also found to be light dependent, with a number of light regulatory elements identified in the promoter region of the gene. In addition, a study was undertaken to demonstrate that the *Arabidopsis AOP2 *gene product is functional *in planta*. The over-expression of a functional *AOP2 *allele was found to successfully convert the precursor methylsulfinyl alkyl glucosinolate into the alkenyl form.

**Conclusions:**

The expression of the *AOP2 *gene has been found to be influenced by light and is most highly expressed in the photosynthetic parts of the *Arabidopsis *plant. The level of *AOP2 *transcript decreases rapidly in the absence of light. *AOP2 *exists as at least three alleles in different *Arabidopsis *accessions and we have demonstrated that one of these, *AOP2-2*, is functionally able to convert methylsulfinyl glucosinolates into the alkenyl form. The demonstration of the *in planta *functionality of the *Arabisopsis AOP2 *gene is an important step in determining the feasibility of engineering glucosinolate profiles in food plants.

## Background

Glucosinolates are a group of secondary metabolites found exclusively in the Plant Kingdom, predominantly within the *Brassicaceae *family. Glucosinolates are substrates for endogenous thioglucosidases called myrosinases. Following tissue disruption, glucosinolates are hydrolysed by myrosinases to produce compounds with a range of biological activities. These activities include the protection of the plant against pathogens and herbivores as well as plant recognition by specialist predators. Glucosinolates are also responsible for the flavour and anti-carcinogenic properties of many *Brassica *vegetables [1-3]. Recent interest has focussed on the regulation of glucosinolate synthesis and the potential for manipulating glucosinolate profiles in food crops [4].

The biosynthesis of glucosinolates occurs in three main stages. Initially the precursor amino acids, such as phenylalanine and methionine may be elongated by the addition of one or several methylene groups. During the second stage the modified precursor amino acids are converted into glucosinolates (glucone formation), and finally secondary modification of the glucosinolate structure takes place [1,2,5,6]. Modifications of the basic glucosinolate molecule create an enormous variety of glucosinolate structures. Secondary modification such as oxidation, hydroxylation, methoxylation, desaturation and glycosylation primarily occurs on the methionine side chain and occasionally on the glucose moiety [3]. The side chain modification of the glucosinolate molecule is of particular importance as the structure of the side chain largely determines the nature of the products formed following glucosinolate hydrolysis by myrosinases [3,6].

**Figure 1:**
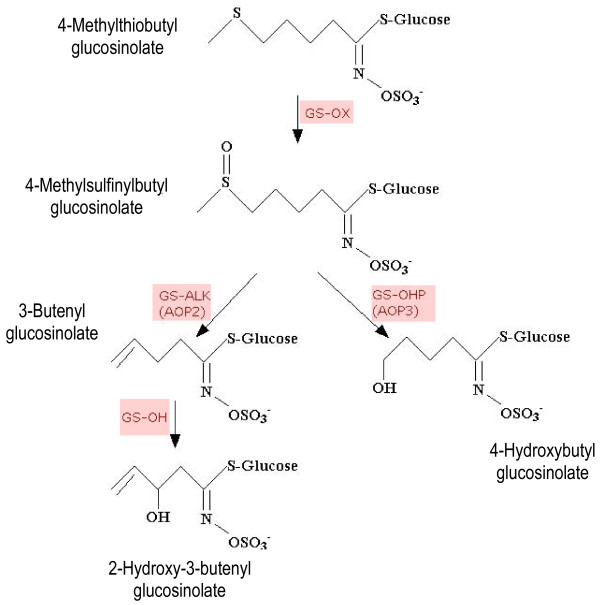
**The modification of the 4-methylthiobutyl glucosinolate side chain **[7,9-11]. Arrows show the steps for 4C aliphatic glucosinolate modification with the locus responsible for each biochemical reaction shown in red text. The structures and chemical names of intermediates and end products are provided.

The major steps involved in 4C aliphatic glucosinolate side chain modification are outlined in Figure 1. Following the formation of methylthioalkyl glucosinolate from methionine, an initial oxidation occurs, catalyzed by the product of the *GS-OX *locus to form methylsulfinylalkyl glucosinolate. Further modification of the side chain is performed by the protein products of the *GS-ALK *and *GS-OHP *loci [7]. In *Arabidopsis *the *GS-ALK *and *GS-OHP *loci map to a region near the top of chromosome four, termed the *GS-AOP *locus [8,9] and fine scale mapping has led to the identification of the three genes, *AOP1, AOP2 *and *AOP3*, that encode 2-oxoglutarate-dependent dioxygenases [10,11]. The *GS-ALK *(*AOP2*) locus protein product is responsible for the formation of alkenyl glucosinolates whilst the *GS-OHP *(*AOP3*) locus protein product is thought to be responsible for the formation of hydroxyalkyl glucosinolates [7]. The alkenyl glucosinolate can also be further hydroxylated through the action of the *GS-OH *locus protein product [7,10,11].

The *AOP1 *gene is believed to be the ancestral gene that gave rise to *AOP2 *and *AOP3 *through a series of gene duplication events. Currently the function of AOP1 is unknown [11]. Heterologous expression in *Escherichia coli *has shown that AOP2 catalyses the conversion of 3-methylsulfinylpropyl- and 4-methylsulfinylbutyl glucosinolates to the corresponding alkenyl glucosinolates, 2-propenyl and 3-butenyl, respectively. The heterologous expression of AOP3 displayed only a weak conversion of 3-methylsulfinylpropyl glucosinolate to 3-hydroxypropyl glucosinolate [11]. However, in the accessions examined a perfect correlation was reported between the expression of a functional AOP2 and AOP3 and the accumulation of alkenyl and hydroxyalkyl glucosinolates, respectively [11]. *Arabidopsis *displays differential *AOP *leaf expression whereby a particular accession expresses either *AOP2 *or *AOP3 *or neither, but not both, and in some *Arabidopsis *accessions the absence of both functional enzymes leads to the accumulation of the precursor methylsulfinylalkyl glucosinolate [11].

The *AOP2 *homologue (*BoGSL-ALK*) from collard (*Brassica oleracea*) has been cloned and characterised and it was found that the gene product is able to catalyse the conversion of methylsulfinylalkyl glucosinolate to the alkenyl form *in planta *[12]. Broccoli, on the other hand, contains a non-functional allele of *BoGSL-ALK *and hence accumulates the 4-methylsulfinylbutyl glucosinolate, glucoraphanin [12]. The accumulation of glucoraphanin is of particular importance as the hydrolysis of glucoraphanin by myrosinase leads to the production of the isothiocyanate, sulforaphane, which is a major inducer of phase II detoxification enzymes that are important in chemoprotection from carcinogens [13].

It is thought that natural variation at the *AOP *locus, in particular the differential expression of the *AOP2 *and *AOP3 *genes, accounts for a large amount of the variation in the glucosinolate profiles and the levels of glucosinolate accumulation found between different *Arabidopsis *accessions [11,14,15]. A mechanism for the differential leaf expression, whereby a particular accession transcribes either *AOP2 *or *AOP3 *but not both, has recently been reported to be due a complete inversion of the *AOP2 *and *AOP3 *structural genes in some accessions, causing the *AOP3 *gene to be expressed from the *AOP2 *promoter [15]. The important role that AOP2 plays as part of a feedback loop which regulates the transcription of other aliphatic glucosinolate biosynthetic genes has also been reported, however, the mechanisms underlying this regulation have not yet been elucidated [16]. Due to the key role that the *AOP2 *structural gene and promoter plays in the aliphatic glucosinolate biosynthetic pathway, the *AOP2 *coding sequence and 5'regulatory region has been characterised in a range of accessions in this report.

The accumulation of glucosinolates has been well studied in *Arabidopsis*. An extensive survey of the glucosinolates accumulated in the *Arabidopsis *accession Col-0 demonstrated that the composition and concentration of glucosinolates varies both developmentally and spatially. It was found that dormant and germinating seeds contain the highest glucosinolate concentration and diversity, followed by inflorescences, siliques, leaves and roots [17]. This pattern of accumulation has also been found in other glucosinolate producing species [18] and correlates with the current theories on the optimal distribution of defence substances which state that the maximum level of protection is provided to the plants' reproductive tissues [19]. In this study the spatial pattern of *AOP2 *transcript accumulation was examined.

The accumulation of glucosinolates in various plants species is also influenced by many environmental factors [20-22]. Climatic and seasonal conditions have long been known to influence glucosinolate concentration in various crop species [23-25]. Glucosinolate levels have also been found to vary throughout the day, with a two-fold change in total glucosinolate concentration being recorded in *B. oleracea *during one diurnal cycle [26,27]. It was found that the concentration of 3-indolylmethyl glucosinolate was reduced in *Brassica napus *seedlings when kept in darkness for 24 hours [28]. It has also been found that watercress (*Nasturtium officinale *R. Br.) accumulates higher levels of the glucosinolate, gluconasturtiin, when grown under long day conditions [29]. Currently little is known about changes in glucosinolate accumulation in response to environmental factors in *Arabidopsis*. In particular, little is known about the environmental factors that activate or repress glucosinolate biosynthetic genes [30]. Therefore a study was initiated to investigate the level of transcript accumulation of the *AOP2 *gene in response to light.

As many plant species within the *Brassicaceae *are important crop plants, the ability to alter glucosinolate profiles *in planta *is potentially valuable from an agricultural perspective. Therefore the current study also aimed to demonstrate that the *Arabidopsis AOP2 *gene product is functional *in planta*. This was done through the introduction of the *AOP2 *gene from the *Arabidopsis *Pitztal accession (*AOP2*_Pi_) into the Columbia accession. It was shown that Columbia plants accumulate methylsulfinylalkyl glucosinolates and it was hypothesised that the introduction of this gene would result in the conversion of the precursor methylsulfinylalkyl glucosinolate to the alkenyl glucosinolate form.

## Results

### *AOP2 *gene expression in the leaves of five *Arabidopsis *accessions

**Figure 2:**
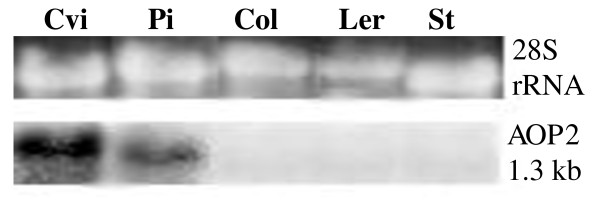
**Northern blot analysis of leaf *AOP2 *expression in five *Arabidopsis *accessions**. The level of *AOP2 *transcript (1.3 kb) in Cvi, Pi, Col, L*er *and St is shown. 28S rRNA is displayed in the top panel to show RNA loading levels in each lane. RNA was extracted from leaf tissue from five plants grown in continuous light.

The accumulation of the *AOP2 *transcript in the leaves of five *Arabidopsis *accessions: Col, Cvi, L*er*, Pi and St, was investigated. Northern blot analysis indicates that of these accessions, Cvi and Pi exhibit high levels of *AOP2 *gene expression, whereas L*er *and St display low or undetectable levels of *AOP2 *gene expression (shown in Figure 2). Figure 2 also indicates that leaf expression of *AOP2 *in Col is not detectable by northern analysis. Expression of a non-functional *AOP2 *transcript in the Col accession has previously been reported [11]. Our inability to detect the *AOP2 *message in the leaves of Col suggests that the gene may be transcribed at a low level and/or the transcript may be unstable.

### Analysis of the *AOP2 *structural gene

**Figure 3:**
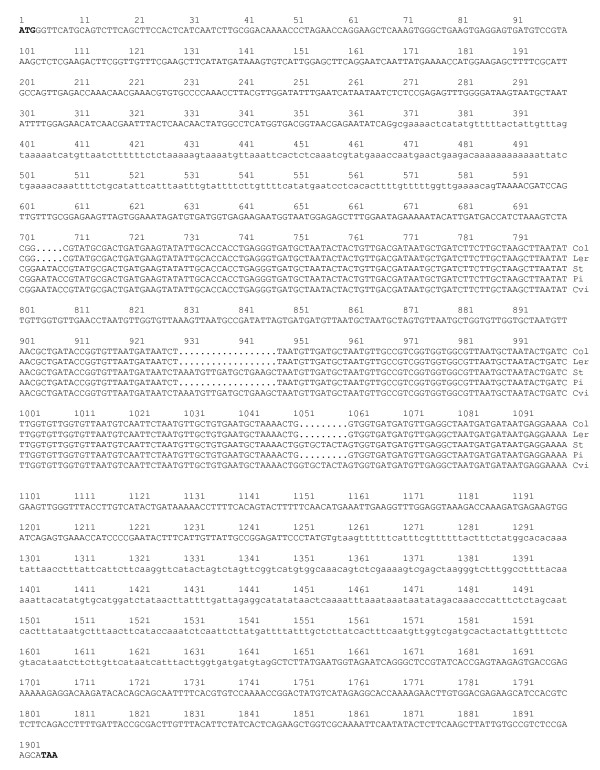
**The *AOP2 *structural gene sequence**. The sequence of the *AOP2 *structural gene in the Col, L*er*, St, Pi and Cvi accessions is shown. The sequences from the five accessions are identical where the sequence is presented as a single line. Exons are shown in upper case letters and introns are shown in lower case letters. The sequence from the five accessions is shown separately in the regions where the three polymorphisms identified in exon 2 (5 bp, 18 bp and 9 bp) are located. Each sequencing reaction was performed three times on different PCR products to ensure accuracy of the sequence.

The *AOP2 *structural gene was sequenced in Col and the two *AOP2*-expressing accessions (Cvi and Pi) and two *AOP3*-expressing accessions (L*er *and St). The sequenced region encompassed all introns and exons between the translation initiating ATG codon and the stop codon. Intron/exon boundaries were predicted from the Cvi sequences using the Genscan program found at (http://genes.mit.edu/GENSCAN.html) and confirmed by sequencing RT-PCR products. The *AOP2 *gene was predicted to contain three exons at positions 1-368, 588-1257, and 1647-1907 bp (Figure 3).

The *AOP2 *gene sequence exhibits complete identity between the five accessions across introns and exons with the exception of three regions in exon two where polymorphisms were found to occur (shown in Figure 3). The *AOP2 *allele present in the Cvi and St accessions was named allele 1 (*AOP2-1*), the Pi allele which contains two polymorphisms (18 bp and 9 bp deletions) was named allele 2 (*AOP2-2*), whereas the Col and L*er *alleles that contain these two polymorphisms as well as an additional 5 bp frame shift deletion was named allele 3 (*AOP2-3*).

The 18 bp and 9 bp polymorphisms cause a nine amino acid difference between the predicted protein product encoded by the *AOP2-1 *and *AOP2-2 *alleles (432 and 423 amino acids, respectively).. If the *AOP2-3 *transcript containing the 5 bp deletion was translated, the frame shift would result in a truncated protein of 172 amino acids being produced, which is in agreement with the Col *AOP2 *gene product (AOP2-3) having been reported as non-functional [11].

Exon 2 from *AOP2 *in twelve additional accessions was amplified and sequenced (data not shown). Six of these accessions (Di-I, Ni, Sf1, Rsch-4, Sf2, Su-O) generated sequence characteristic of the *AOP2-1 *allele, whereas the accessions C24, Can-O, Cond, Sha and Sorbo generated sequence characteristic of the *AOP2-2 *allele, whilst La-O appears to harbour the *AOP2-3 *allele. Northern analyses show that the six accessions analysed containing the *AOP2-2 *allele accumulate *AOP2 *transcript in the leaves, whereas leaf *AOP2 *expression in the three accessions containing *AOP2-3 *could not be detected. The presence of the *AOP2-1 *allele does not correlate either positively or negatively with *AOP2 *expression as four of the *AOP2-1 *containing accessions accumulate leaf *AOP2 *transcripts and four accumulate leaf *AOP3 *transcripts (data not shown).

### *AOP2 *spatial expression profile

**Figure 4:**
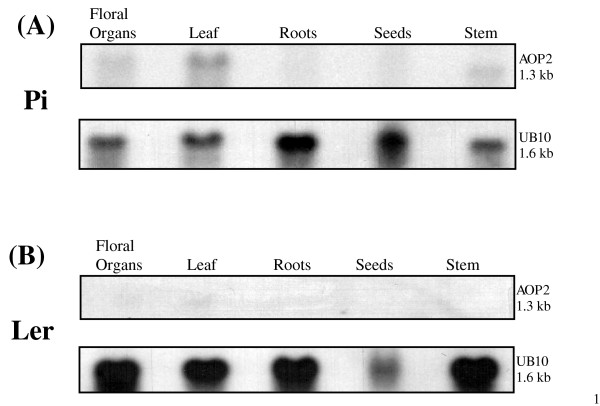
**The spatial accumulation of the *AOP2 *transcript in the Pi and *Ler *accessions**. *AOP2 *expression was examined in floral organs, leaf tissue, root tissue, seeds and stem tissue in Pi (A) and L*er *(B) plants grown in continuous light. The samples were also probed with the *UB10 *gene as a loading control.

An analysis of the *AOP2 *transcript levels in various organs (leaf, stem, floral tissue, roots and dormant seeds) of Pi and L*er *plants grown in CL was conducted (Figure 4a and 4b). Leaf tissue was collected at the four leaf stage of vegetative growth from L*er *and the six leaf stage from Pi. The stem, root and floral tissues were collected from both types of plant approximately four days after the plants had formed floral bolts. In Pi the highest level of the *AOP2 *transcript was detected in the leaf tissue and the transcript was also found to accumulate in stem tissue and to a lesser extent in the floral organs. The root tissue and dormant seeds displayed little or no detectable *AOP2 *transcript. No *AOP3 *expression was detected in Pi plants whereas L*er *accumulated *AOP3 *transcript in the same spatial pattern as that exhibited by *AOP2 *in Pi (data not shown). As expected, no *AOP2 *expression was found in any part of the L*er *accession plant. This indicates that, at least in the plant organs of the accessions tested, the accession-specific leaf expression of the *AOP2 *and *AOP3 *genes extends to the other tissues.

### A comparison of the *AOP2 *5' regulatory region amongst accessions

**Figure 5:**
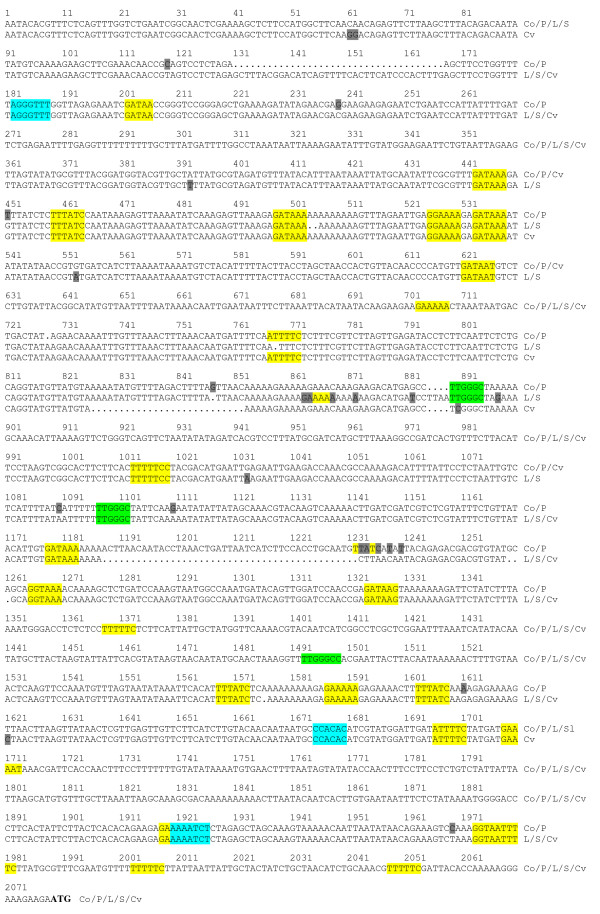
**The *AOP2 *5' regulatory region sequence**. The sequence of the AOP2 5' regulatory region in the Col, Ler, St, Pi and Cvi accessions is shown. The accession to which the sequence pertains is indicated at the end of sequence; Co = Col, P = Pi, L = L*er*, S = St and Cv = Cvi. The sequence changes between the accessions are highlighted in gray. Each sequencing reaction was performed three times on different PCR products to ensure accuracy of the sequence. Putative light and diurnal response elements found in the AOP2 5' regulatory region are highlighted. Yellow; GATA/I box (GATAA) and GT box (GRWAAW where R = A/G and W = A/T): Blue; CBS (AAAAAATCT), ME (CCACAC) and TBX (AAACCCT): Green; FORC^A^/PBX element (T/ATGGGC/ATGGGCC). The translation initiating ATG codon at the end of the sequence is in bold.

Two kb of the 5' regulatory region upstream of the *AOP2 *gene was sequenced with the intention of identifying any promoter differences between *AOP2-*expressing accessions (Cvi and Pi) and Col which could help explain the different expression profiles observed amongst these accessions. The *AOP2 *non-expressing accessions (L*er *and St) were also included in the analyses. Interestingly, the Col and Pi 5' regulatory region sequences were found to be identical to each other (Figure 5), notwithstanding the substantial difference in *AOP2 *transcript accumulation levels in these two accessions. This study has detected no *AOP2 *expression in the Col accession using either northern analysis (Figure 2) or RT-PCR, although it has previously been reported that a low level of *AOP2 *transcript (AOP2-3) is detectable in Col by RT-PCR [11]. This suggests that the *AOP2-3 *transcript, which encodes a non-functional protein, may be degraded rapidly.

Figure 5 shows that the L*er *and St sequences were identical to each other and the Cvi 5' regulatory sequence also shows a high level of similarity to the L*er*/St sequence with only some minor differences being found. The Col/Pi sequence differs substantially from these other three 5' regulatory regions as it contains a 37 bp deletion and 45 bp and 3 bp insertions, as well as a number of single base pair changes throughout the sequence. The comparison between the Cvi and Pi sequences suggests that the major deletion/insertion differences in their 5' regulatory regions is not pivotal in determining the expression profile of *AOP2 *since both these alleles are expressed at comparatively similar levels (Figure 2).

**Figure 6:**
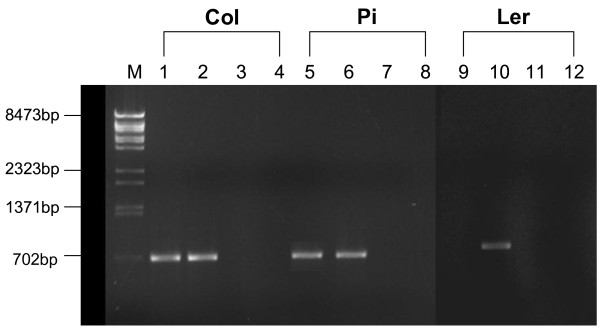
**Gel electrophoresis of PCR-amplified fragments encompassing *AOP2 *and *AOP3 *promoter regions and the structural genes**. PCR reactions, using genomic DNA from the Col, Pi and L*er *as template, were performed with *AOP2 *and *AOP3 *gene-specific primers designed to hybridize at flanking positions in promoter regions and CDS regions approximately 300 bp distal from the ATG translation initiation codon. Lanes 1,5 & 9; products obtained from AOP2 promoter and CDS primers, Lanes 2, 6 & 10; products obtained from AOP3 promoter and CDS primers, Lanes 3, 7 & 11; products obtained from AOP2 promoter and AOP3 CDS primers, Lanes 4, 8 & 12; products obtained from AOP3 promoter and AOP2 CDS primers. Lane M contains λ/BstII size markers.

It has previously been reported that the L*er *accession contains a complete inversion of the *AOP2 *and *AOP3 *structural genes that may have resulted in a promoter exchange. This would suggest that this regulatory region may be driving *AOP3 *leaf expression in L*er *[15]. Gene-specific primers were used to investigate the physical juxtaposition of the *AOP2/3 *regulatory regions and the *AOP2/3 *structural genes in L*er*. The primers were designed to amplify a fragment extending around 300 bp upstream and 300 bp downstream from the initiating ATG codon. Both Col and Pi were included as controls. Amplifications using mixed primers were included in an attempt to detect any *AOP *promoter-CDS linkage anomalies. The mixed *AOP2/3 *primer combinations generated no fragments amongst these accessions (Figure 6). The *AOP3 *promoter-*AOP3 *CDS primer combination generated the expected fragments in the three accessions, whereas the *AOP2 *combination generated a fragment in Col and Pi but not L*er *despite repeated attempts. Sequencing of the *AOP3 *L*er *PCR fragment (data not shown) indicates that in L*er *the proximal 289 nucleotides of *AOP3 *regulatory region is linked to the *AOP3 *CDS (extending 375 nucleotides into the *AOP3 *structural gene). The sequence from the overlapping regulatory region fragments extend this linkage 2 kb upstream indicating that an inversion has not occurred within 2 kb upstream of the *AOP3 *structural gene in the L*er *accession used in this study.

The comparison between the regulatory regions upstream of the *AOP2 *structural gene in the *AOP2*-expressing (Cvi/Pi) and *AOP2 *non-expressing (L*er*/St) accessions found only a small number of exclusive differences, comprised of single and double base pair substitutions and insertion/deletions, and one 4 bp deletion. The majority of these differences are clustered around nucleotides 1184 to 1232 upstream of the ATG (Figure 5; position 847-897). No potential *cis*-acting elements (see below) were identified in this region and it is not clear that these minor changes would explain the substantial difference in the leaf level of AOP2 transcript observed amongst these accessions.

### *AOP2 *expression is regulated by light

Several light-responsive elements were identified in the *AOP2 *5' regulatory region (Figure 5) including GT-boxes and GATA/I-boxes [31-33]. The *AOP2 *promoter also contains a CBS element that is found in the promoters of many clock-regulated genes that have a peak of expression around mid-day [34,35] although Harmer & Kay [36] suggest that CBS does not act alone to specify circadian phase. One ME and one TBX element [37] are also found in the *AOP2 *promoter. These elements, along with flanking sequences are thought to specify the time of day phase and level of transcriptional activity through interactions with photocycles, thermocycles and the circadian clock [37]. Interestingly, the *AOP2 *promoter contains a number of *FORC*^*A *^elements which occur in *Arabidopsis *genes that are up-regulated in response to oomycete and fungal infection [38]. Similar elements (GATA, CBS, TBX &*FORC*^*A*^) are found in the L*er AOP3 *promoter (data not shown). The positions of the elements in the *AOP2 *promoter sequence are highlighted in Figure 5. Sequence polymorphisms between the *AOP2*-expressing accessions (Cvi/Pi) and non-expressing accessions (L*er*/St) lead to the disruption of only one putative GT box response element in the non-expressing accessions at position 767 (Figure 5).

**Figure 7:**
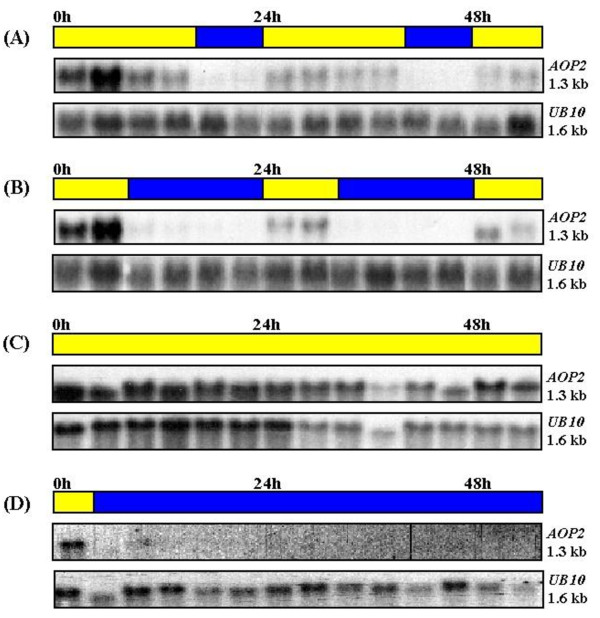
**The accumulation of the *AOP2 *transcript in response to different light regimes**. Pi plants were grown under LD conditions (16 hours light/8 hours dark) and either kept in LD conditions (A) or transferred to SD conditions (8 hours light/16 hours dark) (B) or CL conditions (continuous light) (C) or constant darkness (D). Samples were collected every 4 hours for 56 hours. The northern blots were also probed with the *UB10 *gene as a loading control.

An examination of the microarray data located at "The Diurnal Project" (http://diurnal.cgrb.oregonstate.edu/) shows *AOP2 *exhibiting a very low level of expression in Col that peaks around midday when plants are grown in LD conditions at constant temperature [39]. However, this microarray-based *AOP2 *expression pattern in Col is very similar in both amplitude and phase to that depicted for *AOP2 *in L*er*, which is generally accepted as being an *AOP2 *non-expressor. This contrast with the *AOP3 *expression pattern in L*er *which shows a substantially higher diurnal expression with a maximum at ZT 12 under LD conditions. Since neither of the accessions utilized in "The Diurnal Project" express substantial levels of *AOP2 *transcript, the accumulation of the *AOP2 *transcript in Pi plants under various light conditions at a constant temperature was investigated. The Pi plants, which express *AOP2 *but not *AOP3*, were initially grown under long day (LD) conditions (16 hours light: 8 hours dark) until they had reached the six-leaf stage. The plants were then transferred to either short day (SD) conditions (8 hours light: 16 hours dark), continuous light (CL) or remained under LD conditions. Samples were collected at 4-hour intervals and the transcript level of the *AOP2 *gene was subsequently analysed by northern blot (Figure 7). The *AOP2 *transcript was detectable in plants exposed to light, however it was found that when the plants entered a dark period the expression of *AOP2 *decreased to an almost undetectable level within four hours in both LD (Figure 7A) and SD (Figure 7B) conditions. When plants again entered a light period the transcript level returned to a detectable level within a few hours. Plants that were transferred from LD conditions to CL showed relatively constant expression of *AOP2 *(Figure 7C) whereas no transcript was detectable at all in plants transferred to continuous darkness. Hence no evidence of *AOP2 *transcript oscillations in the absence of environmental stimuli was obtained.

### Constitutive expression of the *AOP2-2 *allele in the Col background alters the glucosinolate profile

Transgenic *Arabidopsis *lines were created in the Col accession. Col expresses low levels of *AOP2 *(Figure 2) and no *AOP3 *transcript. Absence or low levels of AOP2 or AOP3 expression should result in accumulation of methylsulfinylalkyl glucosinolate [11] in the Col accession, whereas the introduction of a functional *AOP2 *gene would be expected to convert methylsulfinylalkyl glucosinolate to the alkenyl glucosinolate form. The *AOP2*_Pi _gene (*AOP2-2*) from the Pi accession driven by the CaMV35S promoter was used to create a 35S:*AOP2*_Pi _line in the Col accession.

**Figure 8:**
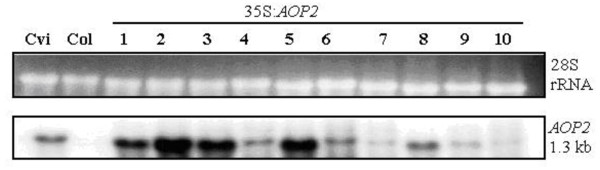
**Northern analysis of the transcript levels of 35S:*AOP2*_*Pi *_in the transformed lines**. Leaf tissue from five T_2 _plants from each independent 35S:*AOP2*_*Pi *_line was pooled and total RNA was extracted. Lane 1 contains total RNA extracted from the leaf tissue of five Cvi plants, which express *AOP2*, and was included as a positive control. Lane 2 contains total RNA extracted from leaf tissue of five wild-type Col plants, and was included as a negative control. Lanes 3 - 12 contains total RNA from the transgenic lines indicated and shows the transcript levels of *AOP2 *in the ten independently transformed lines. The upper panel depicts 28S rRNA stained with ethidium bromide to indicate the level of total RNA loaded in each lane.

Ten independent transformants were obtained. Following confirmation of the presence of the construct by the amplification of the expected 699 bp PCR fragment, the transcript levels from the *AOP2 *gene within those lines was analysed. Northern blot analysis (Figure 8) shows the presence of *AOP2 *transcript in all the appropriate transgenic lines. RNA from an *AOP2 *expressing accession (Cvi) was included as a positive control (lane 1). The inclusion of wild-type Col RNA on the northern blot shows that the low expression of *AOP2 *was not detectable by northern analysis in untransformed Col plants. A degree of variability in the transcript levels of the *AOP2 *gene is observed in the transformed lines.

**Figure 9:**
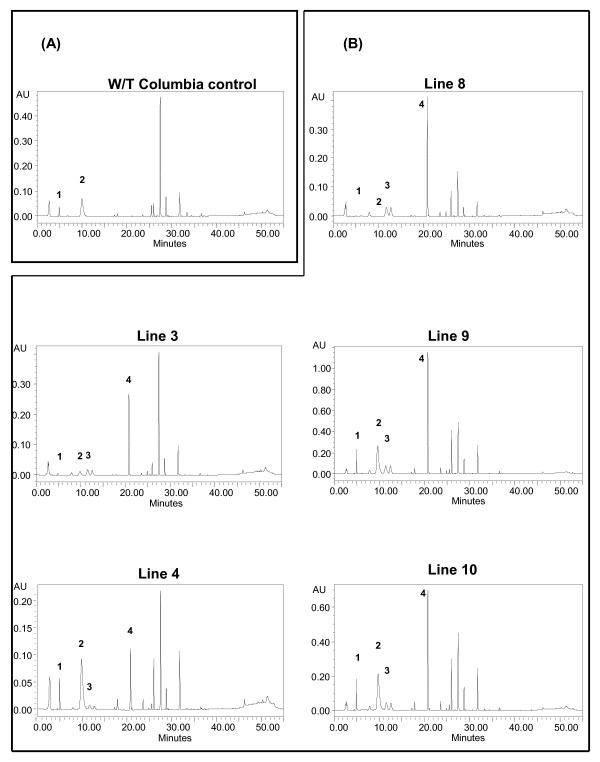
**The typical HPLC profile obtained for wild-type Col (A) and the five 35S:*AOP2 *transgenic lines (3, 4, 8, 9 and 10) (B). Panel A: **The typical HPLC profile of wild-type Col is shown. Peak 1 corresponds to the 3-methylsulfinylpropyl glucosinolate, and peak 2 corresponds to 4-methylsulfinylbutyl glucosinolate. **Panel B: **The HPLC profiles of transgenic lines 3, 4, 8, 8 and 10 are shown. Peak 1 corresponds to 3-methylsulfinylpropyl glucosinolate; peak 2 corresponds to 4-methylsulfinylbutyl glucosinolate, peak 3 corresponds to 2-propenyl glucosinolate peak 4 corresponds to 3-butenyl glucosinolate.

HPLC analysis of the transgenic lines was conducted to determine whether the introduction of *AOP2 *into the Col background alters the glucosinolate profile. Five T_3 _generation homozygous *AOP2 *lines were obtained and used for HPLC analysis. The selected lines were 35S:*AOP2*_*Pi*_: 3, 4, 8, 9 and 10 from Figure 8. Figure 9(A) shows the typical HPLC result obtained for wild-type Col. The identities of the peaks were confirmed using standards and peaks 1 and 2 were found to correspond to the two precursor glucosinolates present in wild-type Col, 3-methylsulfinylpropyl (4.7 mins) and 4-methylsulfinylbutyl glucosinolates (9.9 mins), respectively. The HPLC analysis of Col accession was repeated several times with similar results and corresponds with the profile reported in the literature [11,12].

Figure 9(B) shows the typical HPLC results obtained for the 35S:*AOP2*_Pi _lines 3, 4, 8, 9 and 10. Evidence for the expression of active AOP2 in the transgenic lines was provided by the appearance of two new peaks: 2-propenyl (12.2 mins; peak 3) and 3-butenyl glucosinolate (20.6 mins; peak 4), which were not observed in the wild-type Col control. Line 3 shows a high degree of conversion of the 4-methylsulfinylbutyl glucosinolate precursor to the 3-butenyl glucosinolate. Line 8 showed a similar result, lines 9 and 10 showed slightly less conversion and line 4 showed the least amount of conversion. Lines 3 and 8 also showed almost complete conversion of the 3-methylsulfinylpropyl glucosinolate precursor into 2-propenyl glucosinolate.

## Discussion

Much of the genetic and phenotypic diversity evident in natural *Arabidopsis *accessions occurs as a result of drastic mutations, such as deletions and nucleotide changes resulting in the introduction of stop codons. In some cases these changes occur in several accessions which suggest they may be adaptive [40]. In the present study, three *AOP2 *alleles were identified based on the presence or absence of polymorphisms, which were all located in the second exon. When compared with *AOP2-*1 (the reference allele), the allele, termed *AOP2-3*, was found to contain one 18 bp and one 9 bp deletion polymorphism, in addition to the 5 bp deletion polymorphism previously identified [11]. The Col and L*er *accessions were found to possess the *AOP2-3 *allele and *AOP2 *transcript was not detected in the leaves of either of these accessions. If the *AOP2-3 *allele is transcribed, the 5 bp mutation would cause the introduction of a series of stop codons in the transcript, which may have a negative effect on mRNA stability [41]. It should be noted that the L*er *accession used in the previous study [11] was found not to possess the 5 bp polymorphism in exon 2, whereas the L*er *used in the current study was found to possess the *AOP2-3 *allele containing the 5 bp deletion. The expression of *AOP2 *in the leaves was not detected in the L*er *accession used in either study [11]. In addition, the Pi-0 accession used in the previous study was found to express *AOP3 *instead of *AOP2 *[11], whereas the Pi accession used in the current study displays *AOP2 *leaf expression exclusively. These discrepancies are most likely due to the use of different Pi and L*er *strains. The Pi and L*er *used in the present study are laboratory strains that have been selected over a number of years.

The *AOP2-2 *allele that was found to be transcribed in our Pi accessions does not contain the 5 bp deletion but does contain the two in-frame 18 bp and 9 bp deletion polymorphisms. This would result in a predicted protein product nine amino acids shorter than AOP2 1. These polymorphisms occur in a region of the AOP2 protein that contains several asparagine, valine and alanine rich repeats. Our results, in conjunction with previously reported results [11], indicate that both *AOP2-1 *and *AOP2-2 *encode functional protein products.

The *AOP2-1 *allele was present in both Cvi and St but only expressed in Cvi and not the St accession. St expresses *AOP3 *in the leaves (data not shown). As the St *AOP2-1 *allele does not contain the 5 bp deletion, mechanisms other than mRNA instability arising from a frame shift mutation are likely to be responsible for the lack of *AOP2 *expression in St.

The *AOP2 *5' regulatory region was examined for differences between the *AOP2-*expressing (Cvi/Pi) and *AOP2 *non-expressing (L*er*/St) accessions. No sequence differences in the *AOP2 *5' regulatory regions differentiating the *AOP2-*expressing and *AOP2 *non-expressing accessions were detected within 1 kb of the ATG initiation codon amongst any of these accessions and only a few minor differences were found within the 1 kb analysed further upstream. In addition, none of the major primary sequence differences found in the AOP2-expressing accessions correlate with the level of *AOP2 *transcripts. Hence it is likely that primary sequence differences found in the *AOP2 *promoter regions do not account for the variation in *AOP2 *transcript expression levels amongst the accessions analysed, although reporter gene experiments are needed to confirm this.

Differential *AOP2/3 *expression in the leaves of *Arabidopsis *accessions has been reported as being associated with an *AOP2/3 *structural gene inversion and *AOP2/3 *promoter swap (15). In the L*er *accession, which expresses *AOP3 *in the leaves, such an inversion would be expected to bring the L*er AOP3 *CDS under the control of the *AOP2 *regulatory region. Our results suggest that an inversion has not occurred within the 2 kb upstream of AOP3 in our L*er *accession or within the 2 kb upstream of the AOP2 gene in those accessions analysed that express AOP2. It should be noted that our results do not preclude an inversion/s having occurred outside the regions analysed which may bring the *AOP *regulatory regions under flanking epigentic influences that regulate the spatial expression of the AOP genes. Our inability to obtain a product using the *AOP2-*specific primers on L*er *DNA may indicate a rearrangement has occurred in the *AOP2 *region that affects primer hybridization or amplification. Faint larger bands in the *AOP2 *primer L*er *reaction as well as some bands originating from the use of mixed AOP2/3 primer combinations on other *Arabidopsis *accessions occurred on occasion but these results were difficult to reproduce. Nevertheless the results raise the possibility that a variety of rearrangements may have occurred in the *AOP *region amongst *Arabidopsis *accessions. This warrants further investigation given the implications for glucosinolate production and plant-insect interactions. Other regulatory mechanism differences associated with transcription factor activity or differential mRNA stability may also need to be investigated in order to fully understand the regulation of *AOP *expression.

The spatial expression analysis showed high levels of *AOP2 *transcript were present in the vegetative leaves of Pi. Although there is not necessarily a direct correlation between the site of gene expression and metabolite accumulation, this correlates with reports that aliphatic glucosinolates, in particular C_3 _and C_4 _alkenyl, hydroxyalkyl and methylsulfinylalkyl glucosinolates, accumulate to high levels in *Arabidopsis *vegetative leaf tissue [17,42].

It has been reported that *Arabidopsis *roots accumulate a similar glucosinolate profile to that of rosette leaves, except that they contain a greater proportion of indole glucosinolate [17]. This spatial and temporal analysis of glucosinolates was conducted in the Col-0 *Arabidopsis *accession, which contains non-functional alleles of *AOP2 *and *AOP3 *and thus, does not accumulate alkenyl or hydroxyalkyl glucosinolates [17]. In the current study the *AOP2 *transcript level was analysed in the Pi and L*er *accessions and was found to be at low to undetectable levels in the roots and we have been unable to detect root *AOP3 *in either accession. Therefore it is possible that the lack of a substantial *AOP2 *transcript level would correlate with a lack of alkenyl glucosinolates in *Arabidopsis *roots in general. It is also interesting to note that substantial *AOP2 *expression is only observed in photosynthetic regions of the plant. Thus, it would be interesting to determine whether *AOP2 *transcript is detectable in light grown roots, and if so, whether the change in gene expression would result in the expected alteration in glucosinolate composition. A study has recently been conducted using *Arabidopsis *roots to analyse phenylpropanoid accumulation. It was reported that monolignol glucoside and flavonoid accumulation are induced by light in *Arabidopsis *roots [43]. The response to light was found to require proteins involved in photomorphogenesis and was repressed by proteins involved in skotomorphogenesis. The light-induced accumulation of monolignol glucosides and flavonoids in root tissue was also found to involve changes in the level and cell specificity of phenylpropanoid gene expression [43].

As the levels of many secondary metabolites, such as alkaloids, phenylpropanoids and glucosinolates have been reported to be influenced by light [26,27,44,45] the effect of light on the level of *AOP2 *transcript in the leaves was analysed in the Pi accession throughout a diurnal cycle and it was found that the levels of the transcript varied dramatically. High levels of transcript are observed in light periods, however during dark periods the transcript was found to decrease to an almost undetectable level (Figure 7). This suggests there is a light requirement for *AOP2 *transcript accumulation. This is supported by our spatial analysis. Multiple GT- and GATA/I-boxes were identified in the *AOP2 *5' regulatory region (Figure 5) and these may be important in the light-mediated regulation of the gene. The *AOP2 *5'regulatory region was found to have three main regions containing five, three and two light responsive elements. A high degree of degeneracy has been reported for the GT-boxes [31,33]. Therefore, as there may be additional motifs within the outlined regions that contain GT-boxes that were not detected by the bioinformatics packages, functional analysis will need to be conducted to determine which regions are critical for the light-responsive regulation of *AOP2*. In L*er*, the *AOP3 *transcript also accumulates preferentially in light grown plants (data not shown) although the *AOP3 *transcript does not drop as dramatically in the dark as does the *AOP2 *transcripts in Pi plants.

The *FORC*^*A *^elements found in the *AOP2 *promoter are activators of oomycete- and fungal-pathogen response genes under continuous light, but acts as a repressor under long days and prolonged darkness ([38]. The same element has also been identified as a circadian and diurnal response element, PBX that occurs in many genes associated with protein synthesis [37]. Under hot day/cold night conditions the PBX element confers midnight expression, whereas under photocycles alone PBX confers midday expression. Evrard *et al*. [38] have proposed that these elements, which are conserved throughout the plant kingdom, participate in co-ordinating gene expression associated with photosynthesis, protein production and defence. If *AOP2 *is primarily regulated by these elements, the imposition of thermocycles may phase shift the transcript accumulation cycles. A comparison of the diurnal microarray data for *AOP2 *and *AOP3 *reveals that imposition of thermocycles under long days changes the peak transcript accumulation from around midday (AOP2; ZT = 8, AOP3; ZT = 12) to midnight (ZT = 24) suggesting that the *FORC*^*A *^elements may play a role in regulating *AOP *expression.

The activity of the *Arabidopsis AOP2 *gene has previously been investigated by the heterologous expression of the AOP2 protein in *E. coli *and it was reported that the conversion of the precursor methylsulfinylalkyl glucosinolate to the alkenyl glucosinolate forms is controlled by the *AOP2 *gene [11]. To demonstrate the activity of *AOP2 in planta*, ten independent lines over-expressing the *AOP2 *gene from the Pi accession were created. PCR analysis, designed to amplify a junction fragment spanning the 35S promoter, confirmed that all of the lines contain the appropriate constructs. The northern blot analysis of the ten lines showed expression of the appropriate transgene in all of the lines. The variability in transgene transcript levels in this analysis may be explained by position effects [46], gene copy number [47] and the varying ratios of heterozygous and homozygous T_2 _plants included in the pooled samples analysed.

The glucosinolate profiles in the five 35S*:AOP2*_Pi _over-expressing T_3 _lines analysed by HPLC showed that the *AOP2-2 *allele catalysed the conversion of the two precursor methylsulfinylalkyl glucosinolates to 2-propenyl and 3-butenyl glucosinolates. In the majority of lines tested, the extent of conversion to 3-butenyl glucosinolate was substantial and provides the first demonstration of manipulation of the glucosinolate pathway utilising the activity of *Arabidopsis *AOP2 *in planta. *A previous study has also shown *in planta *modification of the glucosinolate profile in the Col *Arabidopsis *accession by the introduction of the *Brassica oleracea AOP2 *homologue (*BoGLUCOSINOLATE-ALK*). It was found that *BoGLUCOSINOLATE-ALK *is able to convert more than 80% of the 4-methylsulfinylbutyl glucosinolate precursor into 3-butenyl glucosinolate, and the 3-methylsulfinylpropyl glucosinolate precursor into 2-propenyl glucosinolate [12].

## Conclusions

An examination of the *AOP2 *gene across several *Arabidopsis *accessions has revealed that the structural gene encoding AOP2 has three alleles. While one of the *AOP2 *alleles (*AOP2-3*) encodes a truncated protein which is unlikely to be functional, the other alleles (*AOP2-1 *and *AOP2-2*) have now been demonstrated to be functional *in planta*. No major differences where identified in the sequence of the *AOP2 *5'regulatory region between the accessions. *AOP2 *expression in leaf tissue is light regulated and numerous putative light response elements are found within the 5' regulatory region of the *AOP2 *gene. Furthermore, *AOP2 *transcript was found to accumulate predominantly in photosynthetic regions of the plant, with little or no transcript present in root and seeds.

## Methods

### Plant material

The *Arabidopsis *accessions used in this study were either laboratory stocks: Columbia (Col), Landsberg *erecta *(L*er*), Niederenz (Ni); Pitztal (Pi); San feliu 2 (Sf2); Stockholm (St) or obtained from the *Arabidopsis *Biological Resource Center (ABRC): C24 (CS906); Canary Islands (Can-0) (CS1064); Cape Verdi Islands (Cvi) (CS8580); Condara (Cond) (CS6175); Dijon-I (Di-I) (CS1108); Landsberg (La-0) (CS1298); Rschew (Rsch-4) (CS1494); San feliu-1 (Sf1) (CS6855); Shakdara (Sha) (CS6180); Sorbo (CS931); Southport (Su-0) (CS1540).

### Northern blot analysis

The *AOP2 *cDNA (1.3 kb) was digested with the restriction enzymes *Eco*RI and *Hind*III to yield a 300 bp fragment that was used as a probe. The coding sequence of the *Arabidopsis UBIQUITIN10 *(*UB10*) gene was used to quantify gene expression. A *UB10 *probe was obtained by amplification of the *UB10 *gene from genomic *Arabidopsis *DNA using the primers (shown in 5'-3' orientation) GGAAAGACGATTACTCTTGAGG and GTCTTAACGAAAATCTGCATACC.

### Plant growth conditions

Plants were grown in continuous light under gro-lux fluorescent lighting tubes (Sylvania), unless otherwise stated. For light regulation studies Pi plants were grown in LD conditions (16 hours light/8 hours dark) under fluorescent light in glasshouse (GH) conditions until the 6-leaf stage. The plants were then either left in LD conditions or transferred to SD (8 hours light/16 hours dark) or CL conditions (24 hours light) or constant darkness. The temperature was maintained at around 23°C under the different light conditions.

### Amplification and analysis of the *AOP2 *structural gene and 5' regulatory region

All primers were designed based on the published genome sequence of the Columbia *Arabidopsis *accession [48]. The structural *AOP2 *gene was amplified using Pfu polymerase (Promega) using two sets of primers. The first amplification was performed using genomic *Arabidopsis *DNA as template with the following flanking primers (shown in 5'-3' orientation): *AOP2*: TATTGCTACTATCTGCTAACATCTGCAAAC and TTCGTATACCACATGGTTTTTGATAACTTG.

The second amplification was performed using 1 μl of the original reaction as template and the following nested primers (shown in 5'-3' orientation): *AOP2*: ATAGAGCTCTA**GGATCC**GATTACACCAAAAAGGGAAAGAAGAATG and TGAGAGCTCG**ATCTAGA**GAAAGTCGGAATCAAGCGCACACATTG.

The 5' ends of the nested primer pair incorporate the restriction enzyme sites *Bam*HI and *Xba*I, respectively. These sites were subsequently used to sub-clone the 1.9 kb fragment into the pBluescript and pART7 vectors. Internal sequencing of the *AOP2 *gene was performed using the following primers (shown in 5'-3' orientation): *AOP2: *GAGCTTCAGGAATCAATTATGAAAA, GACTAGTATGAACCTTGAAGAATGAATAAA and CGGTAACGAGAATATCAGGTG.

The *AOP2 *5' regulatory region was amplified from *Arabidopsis *genomic DNA in two parts (distal and proximal) using Taq polymerase (Promega). Each fragment was amplified three times to identify random PCR errors. The primers are shown in 5'-3' orientation.

*AOP2 *(distal): AATACACGTTTCTCAGTTTGGTCTG and TTAGAGGAATAAAATGTCTTTTGGC

*AOP2 *(proximal): GCCAAAAGACATTTTATTCCTCTAA and TCTTCTTTCCCTTTTTGGTGTAATC.

Fragments were either sequenced directly as PCR products or were TA sub-cloned into pBluescript and sequenced using the T3 and T7 primers (Promega).

The following bioinformatics packages were utilised in order to identify consensus sequences of potential *cis*-acting elements in the 5' regulatory region of the *AOP2 *gene: **PlantCARE **http://bioinformatics.psb.ugent.be/webtools/plantcare/html/[49]; **PLACE **http://www.dna.affrc.go.jp/PLACE/[50,51] and **AGRIS **http://arabidopsis.med.ohio-state.edu/[52].

### Amplification of fragments linking the *AOP2 *and *AOP3 *structural genes and 5' regulatory regions

The following *AOP2 *and *AOP3 *gene-specific primers were designed to hybridize at flanking positions in promoter regions and CDS regions approximately 300 bp distal from the ATG translation initiation codon. The primers are shown in 5'-3' orientation.

*AOP2 *promoter; ATATAAAATGTGAACTTTAATAG

*AOP2 *CDS; TAAACAATAGTAAAAACATATGAG

*AOP3 *promoter; TATAGAGGTTTATTTTACTGTATC

*AOP3 *CDS; TTTTGGTAAACTTCAAAAGTAGAG

### Preparation of transgenic plants

The *AOP2*_Pi _gene was amplified using Pi genomic DNA as template and ligated into the *Bam*HI and *Xba*I sites of the pART7 vector downstream of the CaMV35S promoter [53]. Following sequence analysis, the pART7:*AOP2*_Pi _construct was digested with *Not*I and the fragment containing the CaMV35S promoter region linked to the *AOP2 *gene was ligated into the binary vector pMLBART [53] and subsequently transformed into *Agrobacterium tumefaciens *strain AGL1. The binary vector pMLBART contains an herbicide (BASTA) resistance gene to aid in the selection of transformed *Arabidopsis *lines. The floral dip method adapted from [54] was used to transform wild-type Col plants. The seeds were collected from these plants and sown in soil. When the seedlings reached the 4-leaf stage they were sprayed twice (three days apart) with the herbicide (0.1% (v/v) BASTA (active ingredient glufosinate-ammonium)/0.1% (v/v) Silwet L-77 mixture). The resulting BASTA resistant seedlings (T_1 _generation) were allowed to self and the T_2 _seedlings were sprayed with BASTA. Tissue from resistant seedlings was further characterised by PCR and northern blot analysis. Seed was also collected from individual BASTA resistant plants and placed under selection to identify homozygous transformed lines. The T_3 _generation homozygous plants were subsequently used in HPLC analysis.

### PCR analysis of transgenic plants

Primers were designed to detect the presence of the 35S:*AOP2*_Pi _construct in transformed *Agrobacterium *and plants. The forward primer (5'-AATGTCAAAGATACAGTCTCAGAAG-3') was designed to hybridise with the 35S promoter and the reverse primers (5'-ATGTTCTCCAAAATATTAGCATTAC-3') was designed to hybridise to the *AOP2 *coding region. The expected fragment size is 699 bp for the *AOP2 *construct.

### Glucosinolate extraction from plant tissue

Glucosinolates were extracted from T3 plants according to the following protocol (A. Leong., personal communication). Plant tissue was freeze-dried and then ground to powder and 150 mg of the powder was then suspended in 5 ml of 70% methanol. If appropriate, glucosinolate standards were added, and the mixture was incubated at 70°C for 20 minutes. The samples were then centrifuged at 3000 rpm for 5 minutes and the supernatant added to a 1 ml column of DEAE-Sephadex A25. The column was then washed twice with deionised water and twice with 1 ml of 0.02 M sodium acetate. Sulfatase solution (75 μl) (prepared as described by [55]) was then added to the column and left to stand overnight. Desulfonated glucosinolates were eluted in 1 ml aliquots of deionised water and analysed by HPLC.

### HPLC analysis of glucosinolates

Glucosinolate samples were analyzed using a Waters (USA) 600E HPLC with a Waters 486 absorbance detector (229 nm). Samples (100 μl) were separated using a Phenomenex Gemini C18 column (150 × 4.6 mm i.d., 5 μm particle size) operated at 1 ml/min at room temperature using the following separation gradient adapted from [56]. Solvent A: H_2_O; Solvent B: MeCN: 1.5 - 5% (v/v) B (6 min), 5 - 7% (v/v) B (2 min), 7 - 21% (v/v) B (10 min), 21-29% (v/v) B (5 min), 29 - 57% (v/v) B (14 min), followed by a cleaning cycle: 57 - 93% (v/v) B (2 min), 5 min hold, 93 - 1.5% (v/v) B (3 min), 6 min hold. Glucosinolate peaks were identified by spiking the samples with the appropriate standards. 2-propenyl glucosinolate was purchased from Sigma-Aldrich. 3-methylsulfinylpropyl glucosinolate, 4-methylsulfinylbutyl glucosinolate and 3-butenyl glucosinolate were kindly supplied by Michael Reichelt (Max Planck Institute for Chemical Ecology, Jena, Germany). The HPLC peaks were analyzed using the Millenium32 software.

## Authors' contributions

DF carried out the gene isolation and initial gene characterization and regulation studies. CN undertook further gene characterization, created the transgenic plants and performed the HPLC analysis. CG performed gene characterization and assisted with the manuscript preparation. AN initiated the experimental program and gave technical and intellectual guidance. All authors contributed to data analysis and interpretation. CN and AN drafted the manuscript. All authors read and approved the final manuscript.
